# A Phase Study of the System: Oxalic Acid/Acetic Acid/Water; Its Significance in Oxalic Acid Crystal Growth^1^

**DOI:** 10.6028/jres.067A.037

**Published:** 1963-08-01

**Authors:** John Strassburger, John L. Torgesen

## Abstract

The presence of limited amounts of water appears to improve the quality of anhydrous oxalic acid single crystals grown from acetic acid solutions. Water concentrations in the saturated solutions which allow crystallization of the anhydrous acid have been determined from the phase study of this ternary system. Near 50 °C the anhydrous acid crystallizes from solutions containing up to 5.2 weight percent water, while the dihydrate appears when water is in excess of this amount.

The phase diagram shows a minimum content of oxalic acid in solution at a solvent composition near 83 percent acetic acid, 17 percent water. The solubility increases with increased acetic acid to the isothermal invariant point, found experimentally at a measured temperature of 50.21 °C to have the composition 20.94 percent oxalic acid, 73.89 percent acetic acid, 5.17 percent water. Decreasing solubility occurs at higher acetic acid concentrations. The maximum water content which allows crystallization of anhydrous acid increases with increasing crystallization temperature. The solubility of oxalic acid in acetic acid/water mixtures at 40° and 50 °C is reported.

## 1. Introduction

Attempts have been made to purify oxalic acid by growth of anhydrous single crystals from its solution in acetic acid. Even when using equipment [1]^2^ with precise control of solution temperture, good crystals only up to 1 cm in length could be grown from glacial acetic acid. Larger crystals became cloudy owing to the inclusion of mother liquor.

The production of anhydrous seed crystals by evaporation of acetic acid at 50 °C is rendered difficult, too, by an excessive nucleation rate resulting in the formation of polycrystalline clusters. However, it has been observed that a small quantity of water, about 5 percent by weight, suppresses the nucleation rate and also produces anhydrous seeds of high quality, more nearly free from inclusions. Water in excess of this amount leads to the formation of oxalic acid dihydrate crystals, which redissolve on further evaporation with the eventual crystallization of anhydrous oxalic acid.

Thus, the indications were that aqueous acetic acid may be the better solvent for the growth of large single crystals of anhydrous oxalic acid. Accurate phase rule data were needed to determine the permissible range of water concentration allowing crystallization and growth of the anhydrous acid. The ternary system oxalic acid/acetic acid/water has consequently been studied as described in this paper.

Hill, Goulden, and Hatton [2] have reported a phase study of the system oxalic acid/sulfuric acid/water at 60 °C. The solution composition at the invariant point is given as 20.5 percent oxalic acid, 44.6 percent sulfuric acid, and 34.9 percent water. This system does not lend itself to the growth of large single crystals of anhydrous oxalic acid because the required concentration of sulfuric acid induces decomposition of oxalic acid in solution. Knox and Richards [3] have measured the solubility of oxalic acid in water solutions of acetic acid. Their work did not extend to concentrations of acetic acid with which anhydrous oxalic acid is in equilibrium.

## 2. Reagents and Apparatus

Reagent-grade oxalic acid (both anhydrous and the dihydrate) and acetic acid were used to prepare all solutions. Assays of the hydrated oxalic acid determined its purity to be 99.97 percent. The acetic acid was assayed at 99.28 percent; the remainder was assumed to be water. No special steps were taken to remove this residual water because it was not essential for the study to include completely anhydrous conditions. The normal laboratory supply of distilled water was used.

**Figure 1:**
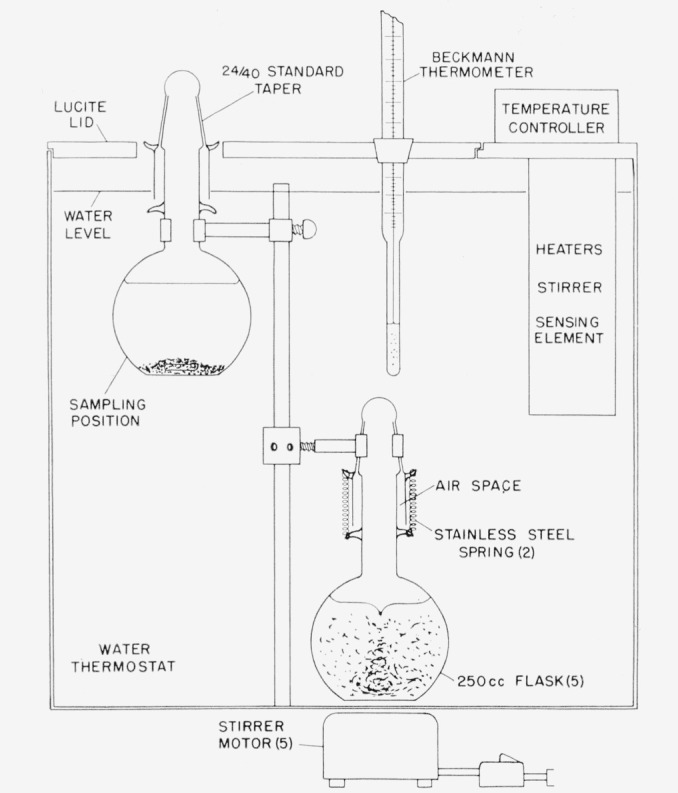
Apparatus assembly for attaining equilibrium solutions.

The various mixtures of the three components were contained in 250-ml Florence flasks modified by attaching inner 24/40 standard-taper seals at the neck. Stopper caps constructed of the outer seal allowed the use of a sealing grease without danger of contamination. Stainless steel retainer springs insured a secure seal beteeen flask and stopper cap and the outer air space, indicated in figure 1, prevented influx of thermostat water.

The solution flasks were supported in a water thermostat of 13 gal capacity. A diagram of the assembled apparatus, shown in figure 1, indicates an upper support position used while sampling the equilibrium mixtures and a lower position used in attaining equilibrium. In the latter case the flasks were clamped around a center post about ¼ in. from the bottom of the bath. A maximum of five flasks could be accommodated in equivalent positions. Each flask contained a Kel-F-coated magnetic stirring bar and continuous stirring was provided by individual magnetic stirring motors mounted under the thermostat below each flask.

The control of thermostat temperature was achieved with equipment of the type developed by Ransom [4], based on the principle of a time-modulated power supply in which the heat applied to the system is in direct relation to the departure of temperature from the desired point. The bulk of the heat was supplied to the bath by a large continuous heater, the sensitive control heat by two Nichrome-wound finger heaters. The bath temperature was observed with a Beckmann thermometer and was controlled to within ± 0.005 °C. The Beckmann thermometer was calibrated with a platinum resistance thermometer which had been previously calibrated at the National Bureau of Standards.

Electrometric titrations were performed with a *p*H meter, Beckman Model G, equipped with external leads.

## 3. Experimental Procedure

### 3.1. Sample Preparation

The saturated solutions were prepared by mixing the appropriate proportion of acetic acid, water, and the excess oxalic acid necessary to saturate the solutions and provide excess solid phase at the desired temperature. The mixtures were heated well above this temperature until complete dissolution of the excess oxalic acid was achieved and then were placed in the water thermostat and allowed to crystallize while being continuously stirred. Saturation equilibrium was achieved by allowing each flask to remain at least overnight in the constant temperature bath. The stirrer of that solution to be analyzed was turned off, and the crystalline material was allowed to settle for a period of approximately 2 hr. The flask was then raised from the bottom and clamped into position near the top of the bath so that only the neck protruded above the water level and through the hole provided in the lid (sampling position in fig. 1). This procedure allowed the solution flasks to be completely immersed in the thermostat until just before sampling.

A pipet was used to remove samples of clear solution which were placed in weighing bottles and stoppered quickly. The pipet was preheated to avoid crystallization while transferring the samples. The slurry was sampled by withdrawing a portion of solid-plus-liquid into a piece of preheated 6-mm glass tubing and transferring it quickly into a weighing bottle. Three samples of the liquid phase and one sample of the solid-plus-liquid mixture were removed. The solution was replaced at the bottom of the water bath, and the procedure was repeated in a day or two for each solution mixture.

Schreinemakers’ “method of wet residues” [5], as described by Ricci [6], was the procedure followed for the determination of tie lines and the composition of the solid phase. The tie lines were determined by analyzing the saturated solution and also a sample of the solid phase mixed with the mother-liquor solution. These two analyses were then points fixing a line joining the liquid and solid phase compositions. Graphical extrapolation of the tie lines to their point of intersection determined the composition of the solid without the necessity of its separation from mother liquor and of its drying preparatory to chemical analysis.

### 3.2. Analysis

Since it was necessary to make a complete analysis on each sample, a procedure was devised to allow compatibility between titration for total acid with sodium hydroxide and titration for oxalate with potassium permanganate. Preliminary analyses showed that acid-base indicators (phenolphthalein or thymol blue) as well as the indicator solvent interfered with the permanganate titration for oxalic acid. For this reason, the samples were analyzed electrometrically for total acid present by titration with sodium hydroxide using a *p*H meter (endpoint= 9.0). The samples were reacidified with sulfuric acid and titrated hot with permanganate for percentage oxalic acid. Percentage water was calculated by difference.

The procedure used for the preparation, storage, and standardization of the sodium hydroxide and potassium permanganate solutions is described by Kolthoff and Sandell [7]. The sodium hydroxide (approximately 0.2*N*) was standardized with NBS standard sample acid potassium phthalate. The permanganate was prepared to be approximately 0.1*N* and was standardized with NBS standard sample sodium oxalate. The analyses of the standard solutions were not considered acceptable until three consecutive determinations gave results which varied by no more than ±0.0001*N*.

## 4. Results and Discussion

**Figure 2:**
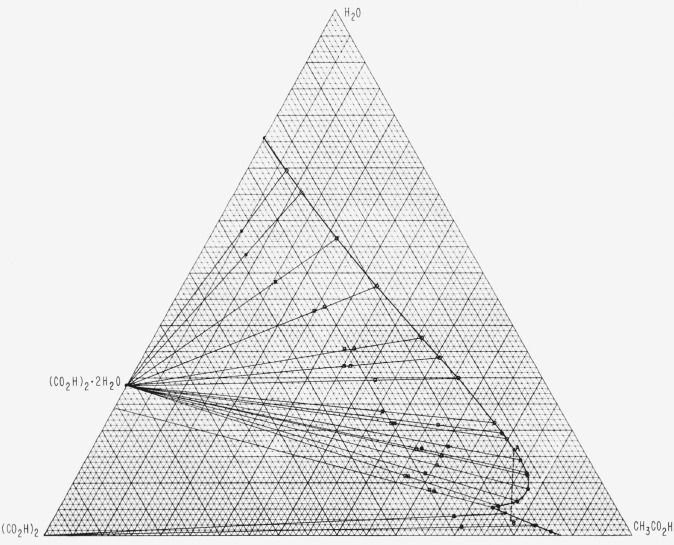
The ternary system: oxalic acid/acetic acid/water; weight percent at 50.21 ± 0.005 °C.

Analytical data at 50.21 °C are given in table 1 and the phase diagram is shown in figure 2. All values are given in weight percent. The experimental values for the liquid-plus-solid analyses are plotted in figure 2 but are not given in table form. The solubility points were found to lie on a smooth curve and the isothermal invariant point was found experimentally to have the composition 20.94 percent (CO_2_H)_2_, 73.89 percent CH_3_CO_2_H, and 5.17 percent water. A minimum solubility of oxalic acid occurs in the region of composition 11.7 percent (CO_2_H)_2_, 73.3 percent CH_3_CO_2_H, and 15 percent water. On the aqueous side of this point, the solubility of oxalic acid increases continuously to a maximum in water alone. On the anhydrous side of the solubility minimum, a rapid increase in solubility occurs until the invarient point is reached, followed by a decrease until completely anhydrous conditions are reached. The tie lines all extrapolated to the theoretical dihydrate or anhydrous composition with good precision, although the large extrapolation from two (or sometimes three) points is not very accurate.

Limited data for the 40 °C isotherm are given in table 2. While the phase relationships at this temperature were not completely explored, the data show the influence of temperature on the crystallization of anhydrous oxalic acid in the presence of water. The maximum water content which allows crystallization of the anhydrous form decreases with decreasing crystallization temperature.

As expected, the solubility of oxalic acid in acetic acid is less than in water. When appropriate corrections are made for the small differences in temperature, the results for the solubility of oxalic acid in water compare with the literature values [8, 9] at least as well as the various literature values compare with each other.

The second set of analyses usually reproduced the first set quite well, showing that equilibrium was reached before the first sampling, and that day to day variability was negligible. If the difference between the mean values of the two individual sets exceeded the standard deviation of a single set, additional analyses were made. Each value in tables 1 and 2, therefore, represents the average of at least six independent determinations. The calculated standard deviations for a single determination, indicating the reliability of each mean value, have the following average values: (CO_2_H)_2_, 0.02; CH_3_CO_2_H, 0.03; water, 0.04.

The phase diagram can be utilized to explain the earlier observations of consecutive crystallization of the dihydrate and anhydrous forms of oxalic acid which occurred during seed growth by evaporation. Published vapor-liquid equilibrium data for the acetic acid/water system, at various temperatures down to 22 °C [10], show that over the entire concentration range the ratio of water to acetic acid is always greater in the vapor phase then in the liquid. We can reasonably assume that a similar relationship holds for the solvent in this ternary system, and that evaporation of the solvent will promote continuous enrichment of acetic acid in the liquid phase.

If a system of acetic acid and water saturated with oxalic acid contains more than 5.2 percent water, as represented by point A (fig. 2) for example, the total composition of the system may be expected to follow a line such as AB as the solvent is evaporated. Evaporation will, in this case, result in crystallization of the oxalic acid dihydrate and its eventual dissolution by the time the dihydrate saturation curve is again reached. Subsequent evaporation will undersaturate the solution with respect to both solid phases until the anhydrous saturation curve is reached, whereafter (upon further enrichment of solvent in acetic acid) anhydrous oxalic acid will crystallize.

## 5. Conclusions

Anhydrous oxalic acid will crystallize near 50 °C from its solution in acetic acid/water mixtures provided the water concentration of the solution does not exceed 5.2 percent. Crystallization of the anhydrous acid at higher temperatures may possibly be achieved over a still greater range of water concentration if necessary. The invariant point of the system at 50.21 °C was found at a solution composition of 20.94 percent (CO_2_H)_2_, 73.89 percent CH_3_CO_2_H, and 5.17 percent water.

## Figures and Tables

**Table 1:** Solubility of oxalic acid in acetic acid/water solutions; 50.21 ± 0.005 °C

(CO_2_H)_2_	CH_3_CO_2_H	H_2_O	Solid phase
			
*wt %*	*wt %*	*wt %*	
24.33	0.0	75.67	H^a^
23.71	6.84	69.45	H
23.14	11.47	65.39	H
21.52	21.90	56.58	H
19. 39	33.09	47.52	H
16.62	45.74	37.64	H
15.53	50.62	33.85	H
14.42	55.61	29.97	H
12.42	66.05	21.53	H
12.10	68.37	19.53	H
11.92	69.85	18.23	H
11.66	74.09	14.25	H
11.89	76.21	11.90	H
11.98	76.56	11.46	H
13.05	78.02	8.93	H
16.12	77.34	6.54	H
19.84	74.62	5.54	H
20.94	73.89	5.17	H+A^b^
19.24	76.66	4.10	A
15.48	82.68	1.84	A
13.31	85.99	0.70	A

a H = (CO_2_H)_2_·2H_2_O.

b A = (CO_2_H)_2_.

**Table 2:** Solubility of oxalic acid in acetic acid/water solutions; 39.94 ± 0.005 °C

(CO_2_H)_2_	CH_3_CO_2_H	H_2_O	Solid phase
			
*wt %*	*wt %*	*wt* %	
17.83	0.0	82.17	H^a^
13.98	36.75	49.27	H
12.29	46.23	41.48	H
11.54	83.73	4.73	H
10.87	88.43	0.70	A^b^

a H = (CO_2_H)_2_·2H_2_O.

b A=(CO_2_H)_2_.

## Footnotes

2 Figures in brackets indicate the literature references at the end of this paper.
